# Editorial: Novel Frontiers in Helminth Genomics

**DOI:** 10.3389/fgene.2020.00791

**Published:** 2020-08-14

**Authors:** Jose F. Tort, Makedonka Mitreva, Klaus R. Brehm, Gabriel Rinaldi

**Affiliations:** ^1^Department of Genetics, Faculty of Medicine, University of the Republic, Montevideo, Uruguay; ^2^Department of Medicine, Washington University School of Medicine, St. Louis, MO, United States; ^3^Institut für Hygiene und Mikrobiologie, Julius Maximilian University of Würzburg, Würzburg, Germany; ^4^Wellcome Sanger Institute, Wellcome Genome Campus, Hinxton, United Kingdom

**Keywords:** flatworm, nematodes, genomics, helminths, neglected diseases

At the brink of the third decade of the twenty-first century, almost a quarter of the world's population are infected by parasitic worms, mainly in deprived areas worldwide. Despite huge and promising efforts from the World Health Organization, there is still much road to cover for the control of these highly prevalent neglected diseases. The current issue highlights how the knowledge of helminth genomics is being translated into novel strategies for diagnosis, control, and the general understanding of parasite biology and adaptation.

Determining infection burdens precisely is critical for surveillance, to evaluate the impact of control programs and to make informed decisions in helminth eradication programs. Two articles in this issue demonstrate how genomics can contribute to these efforts. Grant et al. show that it is feasible to exploit available genomic information to identify species-specific repeated sequences in order to generate qPCR-based diagnostic assays for diverse nematodes. A comparison between these approaches and traditional parasitological methods in soil-transmitted helminths field tests is evaluated and discussed.

Hedtke et al. highlight the relevance of the epidemiological perspective based on genomics in control program decision making. For filarial parasites in Africa, they show that the combination of genomic epidemiology and genome-wide associations could be helpful in defining transmission zones, identifying local or introduced parasites as sources of reinfection and distinguishing genetic markers associated with parasite response to chemotherapy. These are key elements to elaborate surveillance and eradication strategies.

A relevant aspect for these epidemiological approaches is the ability to obtain relevant parasitic samples from natural infections, usually in difficult field conditions where the quantity and quality of samples are limited. Doyle et al. describe the optimization of different methods for extraction and sequencing of high-quality DNA from minimal amounts of parasitic material in several nematodes and trematodes. Although difficult, the authors prove that it is feasible to extract DNA from single parasite eggs and early larval stages, a breakthrough in epidemiological surveillance and genetic population studies, among other relevant applications.

Host-parasite interactions have always caught the attention of parasitologists. It is well-documented that parasites modulate the immune responses of their hosts, but in this issue Martin et al. extend this view by including the gut microbiome. Based on a randomized placebo-controlled anthelminthic trial, the work is the first to analyze changes in the gut microbiome, the presence of parasitic helminths and whole blood cytokine responses in parallel, and opens the avenue to a novel and more complex picture of host-parasite interaction.

The work of Jasmer et al. is a good example of multi-omics approaches applied to understand the complexity of parasitic helminths at the molecular level. They focus on the nematode intestine as a target for therapies, collecting and combining genomics, transcriptomics, proteomics, and miRNA profiling data from representative species of different nematode clades that live and feed on diverse locations within the host digestive tract. The review provides a comprehensive view of the advancements, methods and resources available to analyze gene and protein expression and regulation on the nematode intestine.

Translational research that involves mining available genomic data to understand relevant biological processes is exemplified by the work of Otero et al.. They have taken advantage of available genomes to analyze the basis of alternative mitochondrial electron transport chains used by helminths in hypoxic conditions, showing that although the mechanism seems to be conserved, different gene duplications of the genes involved in the pathway are evident in diverse lineages of both nematodes and flatworms, and evaluated their significance with functional analysis in *C. elegans*.

Reliable methods of functional genomics are needed for analyzing gene function. Lok reviews the methodologies in DNA and RNA transformation in parasitic nematodes, and the seminal works that set proof of principles of CRISPR-CAS9 mutagenesis in strongilyd worms. The value of dominant mutations and regulable Cas9 expression in gene functional validation in parasitic nematodes are thoroughly discussed.

Two articles in this issue are focused on the use of genomics to analyze evolutive aspects of helminths. Compositional features of flatworm genomes are analyzed in detail by Lamolle et al.. Extreme biases in base composition are correlated to profound preferences in codon usages in diverse lineages, with differences between and within free living and parasitic flatworms. The evolutionary implications of these findings are discussed.

By sequencing the tapeworm *Echinococcus oligartus*, and comparing with other species responsible for cystic, multicystic and alveolar echinococcosis, Maldonado et al. provide a novel genomic perspective of cestode evolution, showing that this sylvatic species responsible for unicystic echinococcosis is basal to the genera, and highlighting the relevance of studying sylvatic species.

The use of transcriptomics approaches to shine a light on diverse biological phenomena in helminths is illustrated by works on trematodes. One of the more intriguing aspects in helminth biology is the existence of separate sexes in schistosomes, and the need of pairing for female sexual maturation. While several efforts have focused on the egg-producing female, Lu et al. review the male partner of the couple, highlighting genes related to nervous system and signaling pathways in the complex nature of this interaction.

Helminth parasite survival is supported by massive egg production. Grossi Gava et al. use a combination of RNAi and transcriptomics to demonstrate the involvement of cJun terminal kinases in egg production. Again, using comparative genomics they show that orthologous genes in the nematode *C. elegans* are related to sterility and oocyte maturation.

Regulation of gene expression by diverse RNA mediators is now a central topic in biology, and helminths are no exception. Maciel et al. investigated long non-coding RNAs (lncRNAs) in *S. mansoni* by reanalyzing more than 600 RNAseq libraries. Their extensive characterization shows that lncRNA expression is regulated epigenetically, and they identified sets of correlated gene co-expression modules comprising lncRNA and mRNAs across diverse stages.

In brief, this Research Topic highlights the diversity and richness of genomic-based perspectives used to advance the study of critical aspects of the biology of helminths, that still today inflict much suffering to the most deprived in the world.

## Author Contributions

All authors listed have made a substantial, direct and intellectual contribution to the work, and approved it for publication.

## Conflict of Interest

The authors declare that the research was conducted in the absence of any commercial or financial relationships that could be construed as a potential conflict of interest.

